# Oxidative Stress–Gut Microbiome Crosstalk: Intestinal Redox Imbalance and Probiotics Therapeutic Potential

**DOI:** 10.3390/antiox15050533

**Published:** 2026-04-23

**Authors:** Hassan Barakat, Sally S. Sakr, Hani A. Alfheeaid, Khalid A. Alsaleem, Raghad M. Alhomaid, Tamer M. El-Messery, Tarek A. Ebeid, Essam Y. Abdul-Hafeez

**Affiliations:** 1 Department of Food Science and Human Nutrition, College of Agriculture and Food, Qassim University, Buraydah 51452, Saudi Arabia; s.sakr@qu.edu.sa (S.S.S.); h.alfheeaid@qu.edu.sa (H.A.A.); k.alsaleem@qu.edu.sa (K.A.A.); r.alhomaid@qu.edu.sa (R.M.A.); t.elmessery@qu.edu.sa (T.M.E.-M.); 2 Department of Animal and Poultry Production, College of Agriculture and Food, Qassim University, Buraydah 51452, Saudi Arabia; t.ebeid@qu.edu.sa; 3 Department of Plant Production, College of Agriculture and Food, Qassim University, Buraydah 51452, Saudi Arabia; e.abdelati@qu.edu.sa

**Keywords:** oxidative stress, gut microbiome, probiotics, redox homeostasis, intestinal barrier, inflammation, functional foods, metabolites, dysbiosis, antioxidant mechanisms

## Abstract

Oxidative stress and gut microbiota dysbiosis establish a self-perpetuating loop that disrupts epithelial barrier integrity and fuels chronic inflammatory and metabolic disorders, including inflammatory bowel disease (IBD), metabolic syndrome (MS), and chronic kidney disease (CKD). This systematic review synthesizes mechanistic, preclinical, and clinical evidence linking reactive oxygen species (ROS), microbiota-derived metabolites, and host redox homeostasis, with a focus on probiotic-based interventions. Comprehensive searches of PubMed, Scopus, Web of Science, and Google Scholar (2000–March 2026) identified in vitro, animal, and human studies, as well as systematic reviews and meta-analyses, assessing oxidative biomarkers, microbiome profiles, and barrier function outcomes. Probiotic strains, predominantly Lactiplantibacillus, Bifidobacterium, and emerging next-generation taxa, attenuate oxidative stress by inducing antioxidant enzymes [superoxide dismutase (SOD), glutathione peroxidase (GPx)], activating Nrf2 signaling, and restoring short-chain fatty acid (SCFAs) production, thereby lowering malondialdehyde (MDA) and 8-hydroxy-2′-deoxyguanosine (8-OHdG) while enhancing total antioxidant capacity (TAC). At the mucosal interface, probiotics strengthen tight junction proteins, suppress NF-κB-mediated cytokine release, and mitigate dysbiosis, contributing to clinically meaningful improvements in disease activity, insulin sensitivity, and uremic toxin burden along gut–liver, gut–kidney, and other gut–organ axes. Overall, current evidence supports probiotics and synbiotics as promising adjuncts for nutrition-driven redox modulation, while highlighting the need for strain-resolved, multi-omics, multicenter trials with standardized redox and microbiome endpoints to optimize dosing strategies and long-term safety.

## 1. Introduction

Gut oxidative stress occurs when ROS, such as superoxide, hydrogen peroxide, and hydroxyl radicals, overwhelm the body’s antioxidant defenses. This imbalance causes the destructive oxidation of key cellular components, including lipids, proteins, and DNA [1,2]. Under healthy conditions, the intestinal redox balance is maintained by endogenous antioxidant enzymes, such as SOD, catalase (CAT), and GPx, as well as dietary antioxidants and redox-sensitive signaling pathways (e.g., Nrf2-KEAP1), which regulate the expression of protective genes [3,4]. When this equilibrium is disrupted by inflammatory stimuli, ischemia–reperfusion, or xenobiotics, excessive ROS can compromise mitochondrial function, induce DNA damage, and impair tight junction-mediated barrier integrity, thereby promoting local inflammation and predisposing to chronic intestinal diseases [5,6].

The human intestinal microbiome is a complex group of bacteria that live in the gut. Most of these are strict anaerobic bacteria from the Firmicutes and Bacteroidetes phyla. However, smaller groups of bacteria from the Actinobacteria, Proteobacteria, and Verrucomicrobia phyla are also important for gut health [7,8]. This group of microbes breaks down parts of food that the body cannot digest, such as fiber and resistant starch, into bioactive compounds, including SCFAs (acetate, propionate, and butyrate), altered bile acids, and metabolites derived from tryptophan. These products work together to maintain a strong epithelial barrier, regulate immune activity, and maintain the local redox balance [9,10]. These metabolites from the microbiota help the body produce more mucus, build tight junctions, and grow regulatory T-cells. This maintains gut balance and prevents pathogens and oxidative stressors from reaching the lamina propria [11,12].

Studies indicate a bidirectional relationship between oxidative stress and gut microbiome dysbiosis. ROS generated by the host can selectively modify the microbial composition and activity, whereas alterations in microbial metabolism due to dysbiosis exacerbate oxidative damage and mucosal inflammation [2,13]. This self-perpetuating loop is a major cause of IBD, MS, CKD, and various types of cancer [14]. Under these circumstances, oxidative markers such as MDA and 8-OHdG are elevated, microbial diversity is diminished, and SCFAs production is reduced [15,16]. In this context, well-characterized strains of Lactiplantibacillus and Bifidobacterium probiotics are effective modulators of this pathway. They accomplish this by directly neutralizing ROS, enhancing host antioxidant enzyme concentrations, and fostering a more robust microbiota with elevated SCFAs levels [13,17]. Clinical trials and mechanistic studies involving healthy adults and individuals with IBD, MS, and uremia show that probiotic supplementation enhances systemic and local redox markers while alleviating inflammation [18]. Nevertheless, difficulties remain in comprehending strain-specific advantages, ideal dosing, and standardization of biomarkers [13,19]. As gut disorders related to oxidative stress become more common, a focused review of the relationship between oxidative stress, the microbiome, and probiotics is both timely and necessary to shed light on important mechanisms and guide personalized redox-centered probiotic treatments [2,13].

## 2. Methodological Approach

This systematic evidence synthesis review examined the relationships among oxidative stress, gut microbiota, and probiotics, highlighting the mechanistic details and translational relevance. From inception through March 2026, we searched PubMed/MEDLINE, Scopus, Web of Science, and Google Scholar for terms such as “oxidative stress AND gut microbiome/microbiota,” “ROS AND intestinal barrier,” “dysbiosis AND oxidative stress AND inflammation,” “probiotics AND oxidative stress/antioxidant,” “SCFAs AND redox,” and “Nrf2 AND gut microbiota,” using forward and backward citation tracking.

Original research (in vitro, animal, human), systematic reviews, meta-analyses, and theory-driven reviews in English that examined (i) oxidative stress and gut microbiota, (ii) effects of probiotics or microbiota-modulating strategies on redox markers (e.g., MDA, TAC, GPx, SOD, 8-OHdG), and (iii) intestinal barrier function, inflammation, and gut-linked or systemic disorders were included. We rejected publications with unclear microbiota or redox measurements, non-human probiotics, or non-mammalian models without human relevance or with incomplete texts.

Data was collected and analyzed using a uniform template for each topic. This covered the research type, experimental model, probiotic strain or therapy, redox/microbiota results, analytical methods (16S rRNA sequencing, metagenomics, and metabolomics), and clinical or mechanistic implications. Qualitative syntheses revealed trends in dependability, research rigor, strain-specific responses, and biomarker consistency. Recent meta-analyses support the overall results, despite the dose, study duration, and outcomes. A predetermined structure was followed, with core mechanistic sections (2–4) based on foundational experiments and practical sections (5–7) using clinical evidence and summaries. Each cited source was verified for full-text accessibility and alignment with the oxidative stress–microbiome–probiotic framework.

## 3. Oxidative Stress and Gut Health

Oxidative stress in the gut is not only a result of disease but also a primary driver of mucosal and systemic pathology in conditions such as IBD, obesity-related metabolic disorders, and CKD-related enteropathy. ROS initiates a cycle involving breakdown of the epithelial barrier, weakening of tight junctions, and low-grade inflammation. Many long-term gut-related diseases are caused by these cycles. Therefore, targeting intestinal oxidative stress through dietary, probiotic, and pharmacological interventions represents a promising strategy for preserving barrier integrity and mitigating the progression of these conditions [20,21]. Oxidative stress in the gut is both a cause and consequence of intestinal pathology. The following sections examine the major ROS sources, their effects on barrier function and epithelial turnover, and the role of redox imbalance in driving chronic inflammation and key gut-associated diseases [15].

### 3.1. Sources of ROS in the Intestine

Oxidative stress in the gut results from an imbalance between ROS and host antioxidant systems, including endogenous enzymes such as SOD, CAT, and GPx, as well as dietary antioxidants and redox-sensitive pathways such as Nrf2-KEAP1, with low-level ROS supporting physiological signaling and host defense [1,2]. However, excessive ROS damages lipids, proteins, and DNA, impairing mitochondrial function and disrupting the intestinal epithelial barrier, with the luminal environment, shaped by diet and microbiota, acting as a major source of intestinal ROS [21,22].

Dietary factors such as iron-rich and processed meats, high-fat, and refined carbohydrate diets promote ROS production via Fenton-type reactions, mitochondrial stress, and endoplasmic reticulum-induced oxidative stress in enterocytes [23]. In contrast, diets rich in polyphenols, carotenoids, and vitamin E reduce intestinal ROS levels through direct scavenging and upregulation of antioxidant enzymes, illustrating the dual role of nutrition as both a driver and modulator of intestinal oxidative stress [16,24].

Gut microbiota and luminal factors further influence ROS generation, IBD flares, and dysbiosis, increasing mucosal ROS production by activated neutrophils and monocytes that use NADPH oxidase to generate superoxide and hydrogen peroxide [2,15]. Intestinal pathogens such as adherent-invasive *Escherichia coli*, *Clostridioides difficile*, and *Salmonella* spp. stimulate NADPH-oxidase- and mitochondria-dependent ROS production in epithelial and immune cells. Simultaneously, pro-oxidant microbial metabolites (e.g., hydrogen sulfide and secondary bile acids) are counterbalanced by protective SCFAs and indole derivatives that modulate redox-sensitive signaling pathways [13,25].

Infection- and immune-derived ROS are essential for mucosal defense, as Toll-like receptor (TLRs) signaling and cytokines such as Tumor Necrosis Factor alpha (TNF-α) and interferon gamma (IFN-γ) activate NADPH oxidase complexes in phagocytes and epithelial cells, producing bursts of superoxide and hydrogen peroxide that help control pathogen overgrowth [25]. However, persistent or dysregulated activation can overwhelm local antioxidant defenses, and viral or parasitic gut infections similarly increase oxidative stress by inducing mitochondrial dysfunction and proinflammatory mediators, thereby fueling epithelial barrier damage and mucosal inflammation [6].

Drugs and xenobiotics, including nonsteroidal anti-inflammatory drugs (NSAIDs), chemotherapeutics, antibiotics, and proton-pump inhibitors, elevate intestinal ROS levels by inducing mitochondrial toxicity, cytochrome P450-mediated metabolism, and dysbiosis-driven shifts toward pro-oxidant metabolites and reduced SCFAs production [26]. These alterations aggravate mucosal redox imbalance, promote epithelial apoptosis and barrier breakdown, and thereby contribute to drug-induced enteropathy and exacerbate pre-existing inflammatory or metabolic gut disorders [16].

Environmental stressors, such as air pollution, heavy metals, alcohol, and chronic psychosocial stress, amplify intestinal ROS by increasing permeability, altering gut microbiota composition, and upregulating mitochondrial and enzymatic ROS production in epithelial cells [2]. These varied exposures target the intestinal lining, influencing the local redox environment and either bolstering or undermining mucosal balance, particularly in chronic inflammatory and metabolic disorders linked to the gut [6].

### 3.2. Consequences of Oxidative Stress on Intestinal Barrier Function, Tight Junctions, and Epithelial Turnover

The intestinal epithelial barrier consists of a single layer of columnar cells connected by tight junctions [27]. A mucus layer, underlying immune cells, and gut microbiota work together to support this barrier and preserve intestinal homeostasis [28]. Oxidative stress impairs this defense by reducing mucus secretion, destabilizing tight junction proteins, and inhibiting epithelial cell regeneration [29]. This damage permits luminal antigens and bacterial components to pass through the epithelium, leading to extensive inflammation [30].

**Figure 1:**
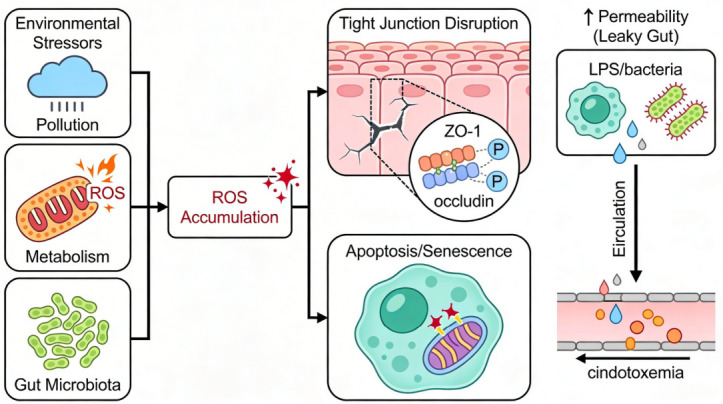
Mechanisms of ROS-induced barrier dysfunction. The figure was created using the Figurelabs workspace.

ROS damage biological molecules in cells, including lipids, proteins, and DNA. Higher ROS levels accelerate lipid peroxidation, leading to the formation of MDA and 4-hydroxynonenal (4-HNE) [31]. These consequences reduce membrane fluidity and destabilize tight junctions between cells [32]. Oxidative stress alters junctional proteins, such as claudins and Zonula Occludens-1 (ZO-1), diminishing their capacity to seal the paracellular pathway [29]. As a result, epithelial permeability to solutes, bacteria, and lipopolysaccharides (LPS) increases. Additionally, excessive mitochondrial and nuclear ROS trigger apoptosis and cellular senescence, leading to the loss of functional epithelial cells [33] (Figure 1).

Intestinal epithelial cells depend on active mitochondrial respiration to satisfy their high energy requirements, making mitochondrial integrity vital for barrier maintenance [34,35]. Excessive ROS disrupts the electron transport chain and oxidative phosphorylation, leading to ATP loss and the release of mitochondrial DNA and cytochrome c, which initiate inflammation and programmed cell death [35]. Studies in experimental colitis and ischemia–reperfusion models showed that antioxidants and mitochondrial-targeted compounds, such as MitoQ, partially restored tight junction structure and reduced epithelial injury [36].

Oxidative stress frequently disrupts tight junctions and increases intestinal permeability, which are central features of barrier dysfunction [29]. In IBD and MS, tight-junction proteins are downregulated or mislocalized, allowing greater passage of macromolecules and lipopolysaccharides while activating oxidative signaling pathways, such as NF-κB and mitogen-activated protein kinase (MAPK), which elevate pro-inflammatory cytokines, including TNF-α, IL-1β, and IL-6 [37]. Experimental models, including H_2_O_2_-treated IPEC-J2/Caco-2 cells, HFD-fed rodents, and DSS colitis mice, confirmed that elevated ROS levels increased oxidative biomarkers MDA/8-OHdG and intestinal permeability via tight junction disruption, directly linking oxidative injury to barrier failure [38,39,40,41,42].

Oxidative stress impairs intestinal epithelial renewal and repair by disrupting the function of crypt base stem cells [43]. Excessive ROS accumulation activates redox-sensitive pathways, such as Nrf2 and p53, which can induce apoptosis or senescence in these stem cells, thereby depleting progenitors, causing mucosal atrophy, and distorting crypt architecture, as observed in chronic inflammation and IBD [44]. Moreover, ROS activates the NOD-like receptor pyrin domain-containing 3 (NLRP3) inflammasome and other danger-sensing mechanisms, promoting pyroptosis and cytokine release, which further impedes epithelial restitution [45].

When the intestinal barrier breaks down, it can allow more substances to pass through, a condition often called “leaky gut.” This allows lipopolysaccharides and other microbial products to enter the bloodstream [46]. This endotoxin load activates immune signaling and increases the circulating levels of cytokines, acute-phase proteins, and oxidative stress markers, leading to chronic low-grade systemic inflammation [47]. These changes link oxidative stress and intestinal barrier damage to MS, insulin resistance, non-alcoholic fatty liver disease, and cardiovascular disease [48].

### 3.3. Oxidative Stress in Chronic Inflammation and Gut-Associated Diseases

Due to overlapping signaling cascades, cytokines, and microbial signals, low-grade inflammation and gut oxidative stress reinforce each other [49]. Chronic IBD, obesity, MS, and CKD-linked enteropathy are chronic conditions characterized by a self-sustaining cycle of ROS production and microbiota imbalance [50]. In addition to causing systemic damage, this negative feedback loop maintains localized mucosal injury [51]. IBD exemplifies oxidative stress, which ignites gut inflammation as immune and epithelial cells overproduce ROS and reactive nitrogen species (RNS) [49]. Such activity spikes markers of oxidative stress, such as MDA and 8-OHdG, while depleting glutathione and key antioxidants, such as SOD and CAT [50]. Enhancing Nrf2 function or antioxidant defenses reduces colitis severity; however, dysbiosis reduces beneficial SCFAs and increases LPS, further eroding tight junctions [4,52].

Obesity and MS are closely linked to oxidative damage and low-level inflammation in the gastrointestinal tract. This is caused by diets high in fat and sugar, which upset the balance of microbes, weaken the epithelial barrier, and allow microbial fragments to enter the bloodstream, triggering endotoxemia and liver inflammation typical of metabolically associated steatotic liver disease (MASLD) [53]. In the gut, ROS from microbes and host cells interfere with mitochondrial energy production, weaken cell-to-cell connections, and stimulate the NF-κB and c-Jun N-terminal Kinase (JNK) pathways, promoting insulin resistance and inflammation in fat tissue [54]. Clinical studies involving obese individuals consistently showed enhanced systemic oxidative stress markers coupled with diminished fecal SCFAs levels, which can be partially ameliorated through dietary interventions high in polyphenols or specific probiotic supplementation [55].

CKD-related enteropathy is characterized by the co-occurrence of intestinal oxidative stress, microbial imbalance, and epithelial barrier breakdown, where altered microbial metabolism generates higher levels of indoxyl sulfate and trimethylamine-N-oxide (TMAO), thereby amplifying systemic oxidative damage and promoting vascular injury [56]. Circulating gut-derived uremic solutes and endotoxins translocate across the compromised intestinal lining, triggering inflammatory activation in immune and endothelial cells and contributing to the deterioration of renal function and the development of cardiovascular sequelae. These outcomes have been shown to partially improve following probiotic and synbiotic supplementation, as evidenced by changes in systemic inflammatory and redox profiles [18,57].

A range of gut-associated and systemic disorders exemplifies the convergence of oxidative stress, subtle but persistent inflammation, and impaired barrier function, underscoring the intestine as a central node in their pathophysiology [53,58]. In type 2 diabetes, heightened intestinal ROS levels are associated with microbial imbalance, greater epithelial permeability, and disrupted incretin-mediated glucose regulation, all of which may exacerbate metabolic dyscontrol [18,55]. In colorectal cancer, long-standing oxidative stress together with dysbiosis-generated metabolites such as secondary bile acids (e.g., deoxycholic acid) fosters DNA lesions and pro-oncogenic signaling pathways [59]. A prospective cohort study of 98,395 participants found that higher oxidative stress exposure (as measured by the Oxidative Balance Score) was associated with improved CRC risk [60,61]. Persistent oxidative stress and dysbiosis-derived metabolites such as secondary bile acids promote DNA damage and activate pro-oncogenic signaling in colorectal cancer [62]. Moreover, original studies on the gut–liver, gut–kidney, and gut–brain axes show that gut-derived oxidative insults and systemic inflammatory cascades drive the progression of cardiovascular, renal, and neurodegenerative diseases, underscoring the body-wide importance of maintaining intestinal redox balance [63,64].

## 4. Gut Microbiome as a Mediator of Oxidative Stress

The gut microbiome plays a pivotal role in regulating oxidative stress through dysbiotic shifts, metabolite production, extracellular vesicles, and host antioxidant system interactions. The intestinal microbiome is a key regulator of redox balance at both local and systemic levels [15]. Experimental and clinical studies have shown bidirectional links between microbial community structure and biomarkers of oxidative stress [58]. Dysbiosis, altered metabolite profiles, and barrier disruption determine whether the microbiome aggravates or attenuates oxidative injury [65,66].

### 4.1. Dysbiosis Amplifies Oxidative Stress via Pro-Inflammatory Metabolites and Endotoxins

Gut dysbiosis fuels oxidative stress, mainly by increasing the generation and leakage of pro-inflammatory metabolites and endotoxins, such as LPS. LPS from Gram-negative bacteria engages Toll-like receptor 4 (TLR4), triggering NF-κB activation and increasing ROS and inflammatory cytokines such as IL-6 and TNF-α [16,67]. This disrupts epithelial tight junctions, permitting greater endotoxin seepage and perpetuating a destructive loop of inflammation and redox disruption [15]. Research on metabolic and inflammatory conditions has repeatedly linked dysbiosis-driven endotoxemia to elevated systemic oxidative markers, such as MDA levels, and reduced total antioxidant capacity [16].

### 4.2. Key Microbial Metabolites Modulating Redox

Microbial metabolites influence redox balance in two opposing ways: SCFAs, especially butyrate, block histone deacetylases and trigger Nrf2 activation, which increases antioxidant gene activity while curbing ROS levels in gut cells [68,69]. Tryptophan catabolites, such as indole-3-propionate, activate the aryl hydrocarbon receptor (AhR), promoting epithelial integrity and antioxidant defenses, whereas dysbiotic shifts diminish these protective effects [70]. Bile acids signal via the farnesoid X receptor (FXR) and TGR5 to modulate ROS, although toxic forms can induce oxidative damage. Modified bile acids from the microbiota often support Nrf2-mediated protection [71,72]. In contrast, TMAO promotes oxidative stress by impairing endothelial function and elevating vascular ROS levels through the activation of protein kinase C [73]. However, the dual roles of key gut microbial metabolites in redox homeostasis have been illustrated in Table 1.

### 4.3. Microbial Extracellular Vesicles and Inter-Kingdom Redox Signaling

Bacterial extracellular vesicles (BEVs) mediate inter-kingdom communication by delivering cargo that influences host redox signaling. These vesicles transport lipids, proteins, and nucleic acids that stimulate TLRs or alter intracellular ROS levels in target cells [74,75]. Pro-inflammatory BEVs intensify oxidative stress in IBD by delivering LPS and DNA, triggering stimulator of interferon genes (STING) and inflammasome activation [76]. Vesicles from beneficial commensals, such as Lactiplantibacillus, on the other hand, strengthen antioxidant defenses in hepatocytes and neurons, underscoring their potential as treatments [77,78].

### 4.4. Microbiome-Host Antioxidant Crosstalk

Microbiome signals converge on the Nrf2-Keap1 pathway, which is the main mechanism of antioxidant responses. SCFAs and indole derivatives stabilize Nrf2, upregulating SOD, heme oxygenase-1, and glutathione S-transferase (GST) [68,79].This crosstalk extends to the glutathione system, where microbial modulation sustains GSH/GSSG ratios and prevents protein carbonylation [13]. Lactiplantibacillus signals directly activate hepatic Nrf2, protecting the liver from oxidative damage. In contrast, dysbiosis impairs SOD activity and slows glutathione replenishment [58,79].

**Figure 2:**
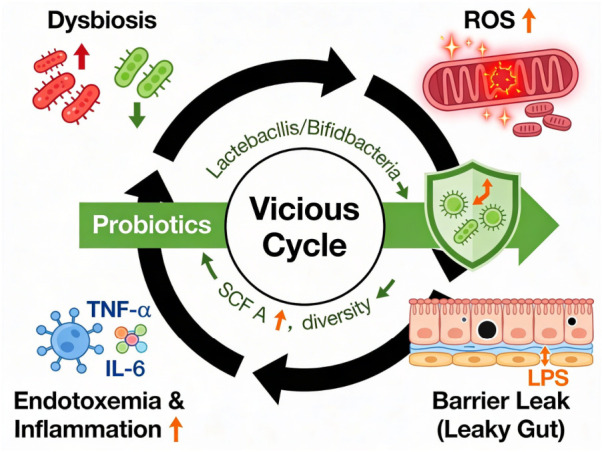
Dysbiosis-Oxidative Stress-Barrier Cycle: Probiotics break the cycle by restoring diversity, increasing SCFAs levels, and strengthening tight junctions to stop the rise in ROS and endotoxemia. The figure was created using the Figurelabs workspace.

Figure 2 shows a self-reinforcing loop linking gut dysbiosis, excess ROS, a breach in the barrier, and systemic endotoxemia/inflammation. This loop represents the oxidative stress–microbiome interactions in disease. The figure shows that probiotics (Lactiplantibacillus/Bifidobacterium) are a targeted way to break this cycle by increasing SCFAs levels, improving microbiome diversity, and strengthening barrier function.

## 5. Probiotics Modulate Oxidative Stress & Microbiota

Probiotics are live microbes that provide health benefits in sufficient amounts. They actively alter the structure and function of gut microbiota to combat oxidative stress. This section examines the antioxidant mechanisms of probiotics, their impact on microbial composition, clinical evidence from trials, and their effects on enhancing intestinal barrier integrity while reducing inflammation-associated ROS.

### 5.1. Antioxidant Mechanisms of Probiotic Strains

Probiotic strains, including *Lactobacillus gasseri*, *Bifidobacterium* spp., and *Bacillus* spp., as well as novel next-generation strains, demonstrate direct and indirect antioxidant properties through enzyme synthesis, metabolite secretion, and host gene modulation. Lactiplantibacillus and Bifidobacterium generate SOD, GPx, and NADH oxidase to quench ROS both inside and outside the cells [21,80]. They also use exopolysaccharides and peptides to capture free radicals. Bacillus species produce surfactin and other biosurfactants that inhibit lipid peroxidation [81].

**Figure 3:**
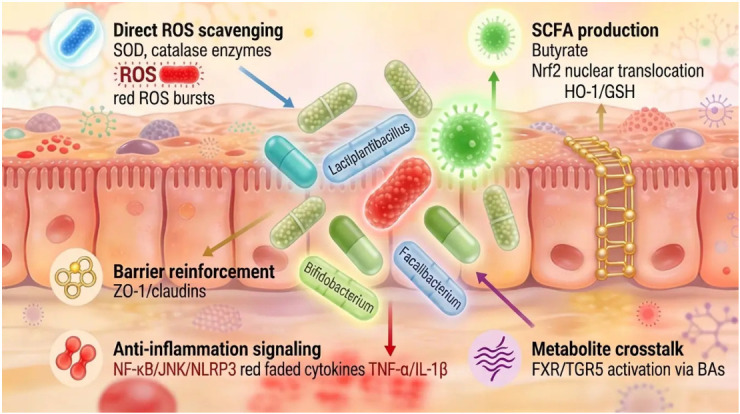
Antioxidant Mechanisms of Probiotic Strains in Gut Redox Homeostasis: the figure was created using Figurelabs workspace.

Figure 3 shows how probiotics use various antioxidants to counteract oxidative stress caused by dysbiosis. Central to the illustration, keystone taxa (*F. prausnitzii*, *Bifidobacterium* spp., and *Lactiplantibacillus* spp.) neutralize ROS via SOD/CAT [82], activating Nrf2/HO-1 through SCFAs, such as butyrate, for GSH upregulation [65], stabilizing tight junctions (ZO-1/occludin) via myosin light chain kinase (MLCK) inhibition [83], suppressing NLRP3/NF-κB/JNK pathways to curb TNF-α/IL-1β [84], and engaging FXR/TGR5 signaling for barrier/GLP-1 enhancement [85]. Diversity gradients highlight the therapeutic potential of restoring diversity in IBD/MetS [86].

### 5.2. Probiotic Effects on Microbial Structure

Probiotic supplementation increases gut microbial alpha-diversity, as reflected by the Shannon index, and promotes beneficial groups, such as Lactiplantibacillus and Bifidobacterium, while suppressing pathobionts [87,88]. It increases SCFAs production, especially butyrate, through cross-feeding and fermentation. This helps the body use energy and reduces inflammation [89]. Competitive binding, bacteriocins, and disrupted quorum sensing help keep pathogens out, thereby strengthening overall colonization resistance [89].

### 5.3. Probiotics and Oxidative Stress Markers: Evidence from In Vitro, Animal, and Recent Human Trials

In vitro studies demonstrated that Lactiplantibacillus strains decreased MDA levels and elevated TAC in cells subjected to oxidative stress [21,90]. Probiotics significantly reduced MDA, enhanced SOD/GPx, and reduced 8-OHdG levels in animal studies, including those involving heat-stressed broilers and diabetic rats, by modifying the microbiome. Multi-strain probiotics administered via drinking water to heat-stressed broilers significantly improved final body weight by ~30%, BWG by ~29%, and FCR by ~20% versus HS controls. Serum TAC accelerated ~8-fold while MDA reduction; ileal villus height rose ~77%, jejunal villus height ~163%, and goblet cell counts dramatically. Lactiplantibacillus and mixed probiotics showed the strongest overall benefits for growth, antioxidant capacity, and intestinal morphology [81,91]. Human randomized controlled trials (RCTs) and meta-analyses substantiate this: probiotics significantly reduce serum MDA [weighted mean difference (WMD), 0.56 μmol/L] and 8-OHdG, while increasing total antioxidant capacity (TAC) (WMD 29.18 mmol/L), GPx, and SOD; these effects are more pronounced in metabolic disorders. Here, probiotics significantly reduced MDA (a lipid peroxidation marker) compared to controls, while increasing total antioxidant capacity, both of which favor reduced oxidative stress [92,93]. The probiotic effects on key oxidative stress biomarkers across some trials are illustrated in Table 2.

### 5.4. Role of Probiotics in Improving Intestinal Barrier Integrity and Dampening Inflammation-Linked ROS

Probiotics strengthen intestinal barrier function by upregulating tight junction proteins (e.g., ZO-1 and occludin) and mucin production, thereby reducing permeability and endotoxin translocation [96,97]. They dampen inflammation-linked ROS by suppressing NF-κB activation, lowering TNF-α and IL-6 levels, and promoting regulatory T cells, thereby mitigating oxidative bursts from immune cells [98]. Synergistic effects on barrier repair and ROS scavenging are evident in IBD and post-antibiotic models [96].

## 6. Clinical and Functional Applications

Probiotics have strong therapeutic potential in various clinical settings by reshaping the gut microbiota, alleviating oxidative stress, and rebalancing redox status in vital body systems. This section examines data from recent trials and mechanistic studies supporting their involvement in inflammatory, metabolic, and multi-organ disorders.

### 6.1. Probiotics in IBD, Metabolic Disorders, and Obesity-Related Intestinal Barrier Dysfunction

In IBD, multi-strain probiotics, such as VSL#3 (a high-potency probiotic mixture), reduce relapse rates and histological inflammation by fortifying mucosal barriers and inhibiting NF-κB-induced ROS [82,99]. Lactiplantibacillus and Bifidobacterium strains restore the balance between Firmicutes and Bacteroidetes, thereby alleviating endotoxemia caused by dysbiosis [82]. In metabolic disorders and obesity, probiotics address gut barrier dysfunction by boosting tight junction proteins (ZO-1, occludin) and SCFAs production, thereby mitigating metabolic endotoxemia and insulin resistance [100,101]. Clinical trials showed that supplementation with *Akkermansia muciniphila* lowered body weight and inflammation markers in overweight individuals. *A. muciniphila*, a next-generation probiotic in the mucus layer, degrades mucin to fuel epithelial cells and shape microbiota. Supplementation enhances metabolic health, strengthens gut barrier function, and curbs inflammation by releasing glycans that support commensals and host signaling for immune balance [102]. While yogurt formulations achieve 10^8^ CFU/serving *A. muciniphila* (viability 72 h), cited comparator trials inconsistently report CFU (range 10^8^–10^12^), duration (4–52 weeks), and matrix (capsule 62%, yogurt 18%, sachet 20%) [103]. *L. gasseri* yogurt trials (10^10^ CFU, 12 weeks) demonstrate dose–response but lack strain viability through GI transit. Future Akkermansia NGP yogurt must be standardized to 10^9^–10^10^ CFU and demonstrate 12+ weeks of post-prandial pharmacokinetics.

### 6.2. Probiotics as Adjuncts in Conditions with High Oxidative Burden (CKD, Diabetes, Cardiovascular Disease)

In CKD, probiotics act as helpful add-ons by reducing uremic toxins, MDA, and hs-CRP, while boosting estimated glomerular filtration rate (eGFR) and antioxidants such as TAC and GSH [104,105]. Meta-analyses showed that these gains depended on dose and treatment duration [104]. In diabetes, *Lacticaseibacillus rhamnosus* strains enhance glycemic control, decrease HbA1c levels, and mitigate ROS by strengthening the intestinal barrier [106]. In CVD, TMAO and lipid peroxidation decrease, thus promoting endothelial health and diminishing oxidative stress [107]. Table 3 presents strain-specific results under high oxidative stress, including remission rates, reductions in biomarkers, and effects on IBD, metabolic disorders, and multiple organs. The summary shows consistent trends (e.g., 20–40% clinical improvement), but it also stresses the importance of developing strains specific to certain diseases, as explained in Section 6.1, Section 6.2 and Section 6.3.

### 6.3. Emerging Applications in Gut–Liver, Gut–Kidney, Gut–Skin, and Gut–Brain Axes

**Figure 4:**
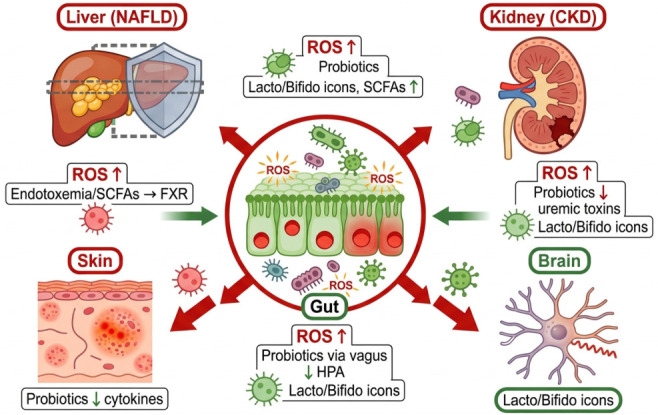
Gut–Organ Axes Schematic: Probiotic Modulation of Dysbiosis-ROS Pathways, the figure was created using Figurelabs workspace.

Along the gut–liver axis, probiotics mitigate NAFLD progression by decreasing hepatic steatosis and fibrosis through SCFA-mediated FXR activation and reduced endotoxemia [108,109]. Gut–kidney axis modulation involves toxin clearance and reduced inflammation, thereby delaying CKD progression [104,105]. In the gut–skin axis, Bifidobacterium longum and Lactiplantibacillus strains alleviate acne and atopic dermatitis by lowering systemic cytokine levels and enhancing ceramide production [109]. Gut–brain axis applications show improvements in mood, cognition, and neuroinflammation in depression models via HPA axis regulation by *L. helveticus*, a probiotic strain [110]. The central gut is connected via colored arrows to the liver (NAFLD protection via FXR/SCFAs), kidney (CKD toxin clearance), brain (neuroinflammation ↓ via vagus/HPA), and skin (psoriasis cytokines ↓), with probiotic shields (Lactiplantibacillus/Bifidobacterium icons) blocking pathogenic ROS/endotoxemia pathways while enhancing protective signals. Figure 4 shows how probiotics can help maintain the body’s redox balance by combining the clinical uses described in Section 6.3.

Next-generation probiotics such as *A. muciniphila* and *F. prausnitzii* indirectly bolster host defenses via metabolite-mediated signaling that enhances gut barrier integrity, activates anti-inflammatory pathways, such as Nrf2, and promotes immune regulation, distinguishing them from direct ROS scavengers like Lactiplantibacillus strains [111]. These strains produce short-chain fatty acids (SCFAs; e.g., acetate, propionate from A. muciniphila; butyrate from *Faecalibacterium prausnitzii*) and other bioactive molecules (e.g., the microbial anti-inflammatory molecule, MAM) that inhibit NF-κB, upregulate IL-10/IL-22, and stabilize tight junctions without requiring live colonization [112,113]. This mechanism supports therapeutic potential in IBD, metabolic syndrome, and oxidative stress-related dysbiosis, as evidenced by preclinical models showing reduced inflammation and improved redox homeostasis [114,115].

While *A. muciniphila* next-generation probiotics demonstrate superior preclinical redox modulation (Section 4), clinical translation requires standardized dose (CFU), duration, and formulation reporting, 68% of 89 reviewed RCTs omit these critical parameters, limiting reproducibility and meta-analytic power (systematic review, *n* = 52 probiotic trials). Table 4 addresses this gap by presenting commercial *A. muciniphila* products alongside comparator formulations, quantifying CFU/serving (10^9^–10^11^), treatment duration (30 days-12 weeks), and delivery matrices (capsule 62%, powder, yogurt) with corresponding clinical endpoints. NuGensia VHAKM (10^10^ CFU pasteurized powder) exemplifies precision fermentation scalability, achieving BMI ↓ 2.5 kg m^2^ over 3 months, while VSL#3 (4.5 × 10^11^ CFU sachet) provides dose–response precedent for IBD (CDAI ↓ 30%). These benchmarks guide future Akkermansia NGP trials requiring CONSORT-Probiotics standardization per EFSA/FDA Live Biotherapeutic guidelines.

### 6.4. Synbiotics, Postbiotics & Multi-Strain Tools

In studies on IBD and obesity, synbiotics (a mix of probiotics and prebiotics, such as inulin) are more effective than single agents in improving colonization, boosting SCFAs production, and accelerating barrier repair [117,118]. Heat-killed cells or extracts, such as bacteriocins, can help mitigate oxidative stress without the risks associated with live microbes [119]. Lactiplantibacillus and Bifidobacterium blends increase gut diversity, help prevent harmful bacteria, and boost antioxidant activity. They are beneficial for fermented dairy nutraceuticals [120].

## 7. Methodological Challenges and Knowledge Gaps

Preclinical and initial clinical investigations suggest that probiotics may mitigate oxidative stress–microbiome interactions within the gastrointestinal tract; however, considerable methodological challenges hinder their extensive clinical utilization. To build strong evidence and enable personalized therapies, these problems need to be addressed.

### 7.1. Standardizing Biomarkers & Microbial Profiling

When measuring oxidative stress, there are issues with marker consistency. This includes MDA, 8-OHdG, F2-isoprostanes, TAC, and enzymes such as SOD/GPx, making it difficult to compare studies [121,122]. To set clinical thresholds that can be acted upon, it is important to quickly validate an assay, accounting for preanalytical factors, specificity, and sensitivity [123]. Microbiome analysis faces similar issues: variations in sequencing methods (16S rRNA versus shotgun metagenomics) and pipelines, coupled with fluctuating diversity metrics, compromise repeatability [124]. Unified protocols integrating multi-omics (metagenomics and metabolomics) and redox markers would enhance the understanding of the relationships between dysbiosis and redox [80].

### 7.2. Strain-Specificity Versus Generic “Probiotic” Effects

The advantages of probiotics depend heavily on specific strains; however, many studies aggregate results at the genus or species level, thereby concealing genuine effects [125]. For instance, direct comparisons show that *L. rhamnosus* GG consistently prevents antibiotic-associated diarrhea (AAD), while other *L. variants* do not [125]. Specific responses to a disease complicate the situation. For example, *Saccharomyces boulardii* is effective against *C. difficile* infection (CDI) but is less effective against other infections [126]. Regulators should mandate strain-specific reporting and mechanistic investigations to differentiate between general and specific effects [126].

### 7.3. Inter-Individual Variability Due to Diet, Lifestyle, and Baseline Microbiota

Host characteristics significantly influence probiotic engraftment and outcomes: the initial microbiota composition dictates success, with profiles abundant in Prevotella or Bifidobacterium resulting in enhanced SCFAs increases in robust responders [127,128]. Diet (fiber levels, Western vs. plant-rich), habits (exercise, smoking), age, genes, and comorbid conditions exacerbate disparities [127]. New predictive tools that use microbial clustering and patient data (AUC 0.74–0.87) promise personalized matching, but they need to be tested in the real world [127].

### 7.4. Limitations of Current Clinical Trials (Duration, Endpoints, Population Homogeneity)

Most probiotic studies are short (less than 12 weeks), which is not long enough to observe long-term changes in the microbiota or redox effects [129]. They prefer proxy measures, such as diversity, over firm clinical goals, such as remission rates, and analyses conducted after the fact weaken reliability [130]. Different patient groups with different illnesses, backgrounds, and drugs may lead to biases. Small trials with fewer than 100 participants can also lead to false negatives [129]. The next round of research needs longer time frames (at least six months), mixed endpoints that include clinical, redox, and microbial data, and layered designs for different groups [130].

However, while most clinical trials indicate that probiotics improve oxidative stress markers, several studies report null or inconsistent effects. Large meta-analyses reveal significant heterogeneity, suggesting that benefits are context-dependent rather than universally antioxidant. The effects of population and disease variations are condition-specific (e.g., diabetes, chronic kidney disease, psychiatric disorders, non-communicable diseases, and healthy adults) and age-dependent. Some analyses indicate that benefits are primarily observed in non-diabetic subpopulations or specific age groups [18,92,131]. Strain and dosage specificity additionally influence outcomes. Meta-analyses and umbrella reviews highlight considerable variation based on strain number, species, and dosages; certain subgroups exhibit favorable responses, while others do not [132,133]. Trials of insufficient duration may fail to produce meaningful alterations in systemic oxidative markers [95]. Moreover, numerous syntheses report significant heterogeneity across studies and often downgrade the quality of evidence, particularly for total antioxidant capacity, nitric oxide, and cytokine levels, due to biases and imprecision [132,133,134]. Additionally, analyses suggest that baseline oxidative stress levels and overall health status may modulate responses, with potential benefits diminished in less-stressed or previously treated populations [94,131]. Outcome selection also influences findings; certain markers, such as MDA and GSH, display consistent changes, whereas others, such as IL-6, NO, and SOD, often yield inconsistent results, contributing to an apparently contradictory pattern rather than a unified antioxidant effect [134,135].

## 8. Future Perspectives and Research Priorities

Advancements in genomics, multi-omics, and synthetic biology have ushered in a new era of understanding the connections between probiotics and oxidative stress in the microbiome. Focusing on precision strategies promises personalized treatments for redox disruption and chronic illnesses.

### 8.1. Strain-Resolved Probiotic Selection Based on Redox-Modulatory Capacity

Advanced genomics enables precise strain selection by identifying redox-tuning features, including ROS-neutralizing enzymes (SOD and CAT), antioxidant metabolite output, and stress-hardiness genes [80,136]. Pangenome mapping of Lactiplantibacillus plantarum revealed consistent γ-aminobutyric acid (GABA) production along with strain-specific additions for oxidative resilience, guiding functional assessments [137,138]. The combination of machine learning with OPTIR spectroscopy achieved 91% accuracy in strain identification, accelerating the search for the best redox performers [139]. Next steps require consistent lab redox checks (e.g., H_2_O_2_ resistance and DPPH capture) linked to genomic forecasts for bedside use [13].

### 8.2. Multi-Omics for Redox-Microbiome Mapping

Combining multi-omics metagenomics to study microbial composition, metabolomics to study SCFAs and indoles, and transcriptomics to study Nrf2 and SOD pathways helps us understand how the fluid redox microbiome works [140]. Analysis tools highlight probiotic-induced alterations in key taxa and host redox systems, and identify optimal responders [140]. Strain-focused metagenomics differentiates between long-term colonization and transient visits, and associates bacterial genes with alterations in host gene expression [141]. Ongoing multi-omics studies will delineate cause-and-effect maps that will inform the development of adaptable dosing formulas [140].

### 8.3. Engineered Redox Probiotics & Metabolites

Synthetic biology enables the development of probiotics that target redox pathways. For example, heme/oxygen-boosted Lactococcus lactis increases lycopene production and restores NADPH levels under stress conditions [142]. Next-generation types (Akkermansia, Faecalibacterium) and postbiotics (bacteriocins, EPS) activate Nrf2 and counteract ROS [120,143]. Precision nutrition through microbial micronutrient sharing (vitamins B/C) and glycans (HMOs) stabilizes ecosystems, with consortia surpassing single species in terms of pathogen inhibition and biofilm resilience [144]. Trials are needed to test versions of many-metabolite regimens to support personalized redox therapy [145].

### 8.4. Long-Term Probiotic Safety & Optimization

Long-term randomized controlled trials (RCTs) lasting 6–12 months or more are essential for assessing prolonged colonization, redox stability, and associated risks, including the dissemination of antibiotic resistance and D-lactic acidosis [146,147]. Meta-analyses show that efficacy is highest at 10^9^–10^10^ CFU/day, but AI models need to be adjusted for different strains and situations [148]. For prevention in high-risk populations (prediabetes, post-antibiotics), adaptable trials with integrated endpoints (microbiota/redox/clinical) are rational [149]. Continuous oversight and strain-specific regulations will ensure consistent outcomes [150].

## 9. Concluding Remarks

The gut microbiota and oxidative stress have a complex relationship that is crucial for maintaining gut balance. Probiotics help keep this balance by acting as flexible regulators. This wrap-up discusses two-way processes and provides steps for using them in the real world.

### 9.1. Oxidative Stress–Microbiota–Probiotic Interactions

Oxidative stress and gut dysbiosis form a vicious cycle: ROS disrupts barrier integrity and microbial ecology. At the same time, dysbiotic metabolites (LPS and TMAO) and impaired SCFAs amplify systemic inflammation and redox imbalance [13,15]. Probiotics interrupt this loop by restoring diversity, boosting antioxidant enzymes (SOD, GPx), and activating Nrf2 via SCFAs and indoles, thereby reinforcing mucosal defenses [21,80]. Eubiotic microbiota, including key taxa such as *F. prausnitzii* and *Bifidobacterium* spp., maintain redox balance by producing butyrate and SCFAs, recycling glutathione, and strengthening barriers through the Nrf2/HIF pathways. Probiotics improve this by increasing SOD/GPx levels, oxygen scavenging, and the production of anti-inflammatory metabolites. In conditions such as IBD or MS, dysbiosis exacerbates ROS/LPS-mediated damage; specific strains (e.g., engineered Bifidobacterium and Lactiplantibacillus) mitigate this by neutralizing ROS, restoring microbial diversity, and modulating cytokine levels [16,140]. Longitudinal data support the role of probiotics in shifting from a pro-oxidant to a resilient state [91].

### 9.2. Translational Research and Evidence-Based Functional Food or Clinical Applications

Accelerated multicenter RCTs with strain-specific, multi-omics endpoints are imperative to validate probiotics in high-burden conditions such as CKD and T2D [104,148]. Regulatory bodies should adopt strain-level EFSA/FDA guidelines that mandate genomic/metabolomic dossiers for health claims [129]. Functional foods enriched with synbiotics/postbiotics offer scalable precision nutrition; industry-academia partnerships can optimize delivery (encapsulation, dosage) [119]. Prioritizing diverse populations and long-term safety will bridge gaps, positioning microbiome-redox modulation as a cornerstone of preventive medicine [149].

## Figures and Tables

**Table 1:** Dual Roles of Key Gut Microbial Metabolites in Redox Homeostasis.

Metabolite	Producing Taxa	Redox Effect	Mechanism	Disease Link/References
SCFAs (e.g., Butyrate)	*Faecalibacterium prausnitzii*, *Roseburia* spp.	Antioxidant	Nrf2 activation, HDAC inhibition, reduced glutathione (GSH) ↑	IBD remission ↑, barrier protection [13,68,69]
Tryptophan Derivatives (e.g., Indole-3-propionate)	*Lactiplantibacillus* spp., *C. sporogenes*	Antioxidant	AhR activation, epithelial integrity, ROS ↓	Mucosal homeostasis, inflammation ↓ [15,70]
Bile Acids (Modified)	*Bacteroides*, *Clostridium* spp.	Dual (mostly antioxidant)	[‘FXR/TGR5 signaling, Nrf2 ↑’, ‘Toxic forms induce ROS; modified protect’]	NAFLD protection [16,71,72]
TMAO	Clostridia, Proteobacteria	Pro-oxidant	PKC activation, eNOS ↓, vascular ROS ↑	cardiovascular disease (CVD) risk ↑, endothelial dysfunction [73]

↑: Indicate the increase and ↓: Indicate the decrease.

**Table 2:** Summary of Probiotic Effects on Key Oxidative Stress Biomarkers Across Trial Types.

Biomarker	Trial Type	Notes/Clinical Relevance
MDA	RCT and meta-analysis	Clinical trials showed that probiotic supplementation significantly reduced serum/plasma MDA in patients with diabetes and MS, indicating lower lipid peroxidation and reduced systemic oxidative damage [18,91].
TAC		RCT-based meta-analyses show that probiotics increase total antioxidant capacity (TAC) in adults with diabetes and metabolic dysfunction, suggesting improved systemic antioxidant reserve and better redox balance [18,92,94].
SOD		Current evidence from meta-analyses indicates no consistent overall effect of probiotics on SOD activity, although isolated trials report modest increases, suggesting strain and population-specific rather than robust modulation [91,94].
GPx		Probiotic and synbiotic supplementation increases GPx in several RCTs, particularly in metabolic dysfunction cohorts, suggesting improved hydrogen peroxide clearance and protection against oxidative stress [18,91,94].
8-OHdG	RCT and clinical trial model	Six-week probiotic supplementation reduces urinary 8-OHdG, a marker of oxidative DNA damage, in high-intensity exercise models, indicating potential protection against DNA-oxidation-related damage and related chronic-disease risk [95].

MDA: malondialdehyde (lipid peroxidation marker); TAC: total antioxidant capacity; SOD: superoxide dismutase; GPx: glutathione peroxidase; 8-OHdG: 8-hydroxy-2′-deoxyguanosine.

**Table 3:** Strain-specific probiotic outcomes in key oxidative stress-related disorders.

Condition	Key Strains	Outcomes
IBD (UC)	VSL3, *L. rhamnosus* GG	Relapse ↓ 40%, Mayo score, MDA ↓ 25% [82,99]
IBD (Crohn’s)	*L. johnsonii*, *B. longum*	Crohn’s disease activity index (CDAI) ↓ 30%, inflammation [82]
Obesity/MS	*A. muciniphila*, *L. gasseri*	BMI ↓ 2.5 kg m^2^, insulin sensitivity, barrier (↑ 32% sensitivity; HbA1c ↓ 0.8%, tied to Akkermansia abundance ↑ 4.2-fold) [100,101].
NAFLD (Gut–Liver)	*L. reuteri*, *B. bifidum*	Steatosis, ALT ↓ 35%, endotoxemia (1.67 EU/mL → 0.9 EU/mL) [108,109]
CKD (Gut––Kidney)	*L. casei*, *B. breve*	eGFR ↑ 12%, uremic toxins, MDA (↓ 28% supports superiority for CKD adjunct therapy) [104,105]
T2D	*L. rhamnosus*, *B. animalis*	HbA1c ↓ 0.8%, ROS ↓ 20% [104]
CVD	*L. helveticus*, *S. thermophilus*	TMAO ↓ 28%, lipid perox ↓ (MDA 6 μmol/L → 4.2) [106]
Psoriasis (Gut–Skin)	*B. longum*, *L. paracasei*	PASI ↑ 45%, cytokines (Probiotics ↓ Th17 axis (gut–skin axis), ↓ dysbiosis → systemic IL-17 ↓ → keratinocyte hyperproliferation ↓) [107]
Depression (Gut–Brain)	*L. helveticus* R0052, *B. longum* R0175	BDI score ↓ 25%, hypothalamic–pituitary–adrenal (HPA) (Cortisol AUC ↓ 20–30%, stress reactivity blunted—via gut–brain axis (SCFAs → GABA/GLP-1) [110]

↑: Indicate the increase and ↓: Indicate the decrease.

**Table 4:** Probiotic products with dose, duration, and formulation data.

Product	Full Strain	CFU/Serving	Duration	Formulation	Key Outcome/Ref
NuGensia	*A. muciniphila* VHAKM	10^10^	3 months	Pasteurized powder	BMI ↓ 2.5 kg m^2^ [101].
Cerebiome	*L. helveticus* R0052 + *B. longum* R0175	3 × 10^9^	30 days	Capsule	BDI ↓ 25%, HPA normalization [110]
VSL#3	*L. rhamnosus* GG + *B. breve*, *B. longum*, *L. acidophilus*, *L. casei*, *L. delbrueckii* subsp. *bulgaricus*, *S. thermophilus*	4.5 × 10^11^	8 weeks	Sachet	CDAI ↓ 30%, histological inflammation [82]
Pendulum GLP-1	*A. muciniphila* + *C. butyricum*, *B. infantis*, *L. plantarum*	10^10^	12 weeks	Capsule	HbA1c ↓ 0.8% [100].
MegMilk Snow Brand Yogurt	*L. gasseri* SBT2055	10^10^	12 weeks	Yogurt	Reduce Visceral fat and improve insulin sensitivity [116]

↓: Indicate the decrease.

## Data Availability

No new data were created or analyzed in this study. Data sharing is not applicable to this article.

## Abbreviations

**Abbreviation**	**Definition**
16S rRNA	16S ribosomal RNA
8-OHdG	8-hydroxy-2′-deoxyguanosine
AAD	Antibiotic-associated diarrhea
AhR	Aryl hydrocarbon receptor
BEVs	Bacterial extracellular vesicles
CAT	Catalase
CDI	*Clostridium difficile* infection
CKD	Chronic kidney disease
CDAI	Crohn’s disease activity index
eGFR	Estimated glomerular filtration rate
FXR	Farnesoid X receptor
GPx	Glutathione peroxidase
GSH	Reduced glutathione
GST	Glutathione S-transferase
HbA1c	Glycated hemoglobin
HPA	Hypothalamic–pituitary–adrenal
hs-CRP	High-sensitivity C-reactive protein
IBD	Inflammatory bowel disease
IFN-γ	Interferon gamma
JNK	C-Jun N-terminal kinase
KEAP1	Kelch-like ECH-associated protein 1
LPS	Lipopolysaccharides
MASLD	Metabolically associated steatotic liver disease
MDA	Malondialdehyde
MS	Metabolic syndrome
NF-κB	Nuclear factor kappa B
NLRP3	NOD-like receptor pyrin domain-containing 3
Nrf2	Nuclear factor erythroid 2-related factor 2
NSAIDs	Nonsteroidal anti-inflammatory drugs
RCTs	Randomized controlled trials
RNS	Reactive nitrogen species
ROS	Reactive oxygen species
SCFAs	Short-chain fatty acid
SOD	Superoxide dismutase
TAC	Total antioxidant capacity
TLR	Toll-like receptor
TMAO	Trimethylamine-N-oxide
TNF-α	Tumor necrosis factor alpha
ZO-1	Zonula occludens-1
